# Transition Phase Regulator AbrB Positively Regulates the *sip1Ab1* Gene Expression in Bacillus thuringiensis

**DOI:** 10.1128/spectrum.00075-21

**Published:** 2021-07-28

**Authors:** Xinxin Shen, Qingyue Yu, Huanhuan Liu, Jiaojiao Wang, Ruibin Zhang, Qi Peng, Fuping Song

**Affiliations:** a College of Life Science, Northeast Agricultural University, Harbin, China; b State Key Laboratory for Biology of Plant Diseases and Insect Pests, Institute of Plant Protection, Chinese Academy of Agricultural Sciences, Beijing, China; College of New Jersey

**Keywords:** AbrB, *Bacillus thuringiensis*, *sip1Ab1*, transition phase regulator

## Abstract

Bacillus thuringiensis secreted insecticidal proteins (Sip) are a secretion that is toxic to coleopteran pests. However, the transcriptional mechanism of *sip* genes is still unknown. The transcriptional regulation of the *sip1Ab1* gene and the expression of the Sip1Ab1 protein were investigated in this study. The results demonstrated that the secretion of the Sip1Ab1 protein in HD73 was almost the same as that in the original QZL38 strain during the transition phase. Analysis of the β-galactosidase activities of *sip1Ab1-lacZ* in both the HD73 and *abrB* mutant strains indicated that the transcription of *sip1Ab1* is dependent on AbrB. Electrophoretic mobility shift assays showed that AbrB could bind with the *sip1Ab1* promoter, and two binding sites of AbrB in the region of the promoter of *sip1Ab1* were determined by DNase I footprinting assays. All of the above-described results proved that AbrB positively regulates the *sip1Ab1* gene.

**IMPORTANCE** Bacillus thuringiensis Sip proteins are secreted insecticidal toxins that are toxic to coleopteran pests. In this study, we investigated the transcriptional mechanism of the *sip* gene and showed strong evidence that Sip1Ab1 is secreted in the transition phase and that AbrB, a transition phase regulator that is usually a repressor, positively and directly regulates *sip1Ab1*. Reports of AbrB positive regulation are rare, even in Bacillus subtilis. To the best of our knowledge, no toxic gene has been reported to be positively regulated by AbrB in Bacillus species.

## INTRODUCTION

Bacillus thuringiensis is a Gram-positive, spore-forming bacterium that can be classified in the Bacillus cereus group (1). It is characterized by the formation of parasporal crystal proteins and spores during the stationary phase of its growth cycle (2). These proteins possess highly specialized insecticidal activities against numerous insect species, including members of Lepidoptera, Coleoptera, and Diptera (3, 4). Some B. thuringiensis strains can secrete proteins during the vegetative growth phase. These secreted proteins are designated vegetative insecticidal proteins (Vip) and secreted insecticidal protein (Sip), which have insecticidal activity and extend the overall host range (5–7).

Only two kinds of Sip proteins, which are mainly secreted by vegetative cells, have been found thus far (8). Donovan first discovered the Sip1Aa1 protein (encoded by *sip1Aa1*) in strain ED2158 and studied its insecticidal activity against *Coleoptera* insects. Sip1Aa1 can cause tobacco aphids, cotton bollworm larvae, and maize root leaf beetles to shrink and lose weight (8). It has a lethal or growth-inhibiting effect on Leptinotarsa species, Diabrotica undecimpunctata howardi, and Diabrotica virgifera virgifera. Another gene, which is highly similar to *sip1Aa1*, was initially obtained from B. thuringiensis strain QZL38 and named *sip1Ab1*. Sip1Ab1 showed insecticidal activity against Colaphellus bowringi Baly (9). Sip1Aa1 and Sip1Ab1 both exhibit typical predicted Gram-positive consensus secretion signals at the 30th amino acid (10), and they also share 46% similarity to the 36-kDa Mtx3 mosquitocidal protein (ETX_MTX2 protein family) (8, 11). However, the regulatory mechanism of Sip protein expression is still unknown.

In Bacillus subtilis, AbrB is a global regulatory factor that regulates gene transcription in the log phase or transition phase (12). Purified AbrB protein binds specifically to fragments of DNA containing the promoters it affects (13). AbrB directly regulates more than 100 genes and influences hundreds more indirectly (14, 15). AbrB mainly functions as a transcriptional repressor for gene transcription, but it also acts as an activator (16) involved in biofilm formation, antibiotic production, capacity development, extracellular enzyme production, motility, and sporulation (17–22). Some examples of positive regulation of AbrB have been reported, such as *scoC*, *rbs*, and *citB* genes (16, 17, 23). Bacillus anthracis AbrB negatively regulates the toxin genes *pagA*, *lef*, and *cya*, which have higher transcriptional activity in the log phase (24), but the regulation is not direct (25).

The Sip1Ab1 proteins in strain QZL38 and their heterologous expression in HD73 were analyzed in this study. The transcription activities of the *sip1Ab1* gene promoter in the HD73 wild-type strain and *abrB* mutant strain were analyzed using β-galactosidase. Electrophoretic mobility shift assays and DNase I footprinting assays showed the relationship between AbrB and the *sip1Ab1* promoter.

## RESULTS

### Sip1Ab1 is secreted in the transition phase.

**FIG 1:**
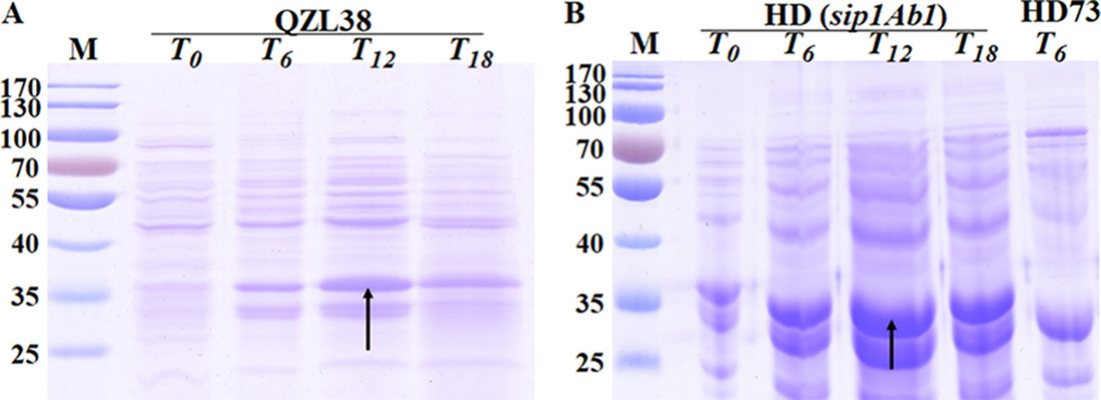
Sip1Ab1 protein expression in strains QZL38 and HD (*sip1Ab1*) in LB medium. (A) Sip1Ab1 protein expression in supernatants of strain QZL38. Lane M, protein marker 26616. Lanes 2 to 5 show the four periods ending at *T*_0_, *T*_6_, *T*_12_, and *T*_18_, respectively. (B) Sip1Ab1 protein expression in supernatants of the strain HD (*sip1Ab1*). Lane M, protein marker 26616. Lanes 2 to 5 show the four periods ending at *T*_0_, *T*_6_, *T*_12_, and *T*_18_, respectively. Lane 6 shows the HD73 wild-type strain in the period ending at *T*_6_.

In order to clarify the expression of the Sip1Ab1 protein in strain QZL38 and its heterologous expression in strain HD73, the promoter and open reading frame (ORF) of *sip1Ab1* were ligated into the pHT304 vector and then electroporated into strain HD73. Time zero (*T*_0_) was defined as the end of the exponential growth phase. The forespore septum was formed at 13 h after *T*_0_ (*T*_13_) (26). *T*_0_ to *T*_13_ covers the transition phase in LB medium of the HD73 strain. The same volume of the culture supernatants at *T*_0_, *T*_6_, *T*_12_, and *T*_18_ in LB medium were collected and concentrated to 2 ml using a dialysis bag. The same loading volumes of strains QZL38 (Fig. 1A) and HD73 (Fig. 1B) bacteria in the culture supernatant were subjected to SDS-PAGE analysis. Through analysis of the protein characteristics and protein molecular weight, the protein bands indicated by the arrows in the figure were identified by mass spectrometry (see Materials and Methods) and were confirmed to be the Sip1Ab1 protein. The Sip1Ab1 protein had the highest expression at *T*_12_ in strain QZL38, and was heterologously expressed in strain HD73. This result suggested that the Sip1Ab1 protein was regulated and expressed during the transition phase. This also means that the transition phase regulator may be involved in the expression of the *sip1Ab1* gene. When we selected some global regulatory factors, namely, Spo0A, SigH, CcpA, and AbrB, and tested the regulatory relationship with *sip1Ab1*, only AbrB had a regulatory effect.

### AbrB positively regulates the *sip1Ab1* gene.

**FIG 2:**
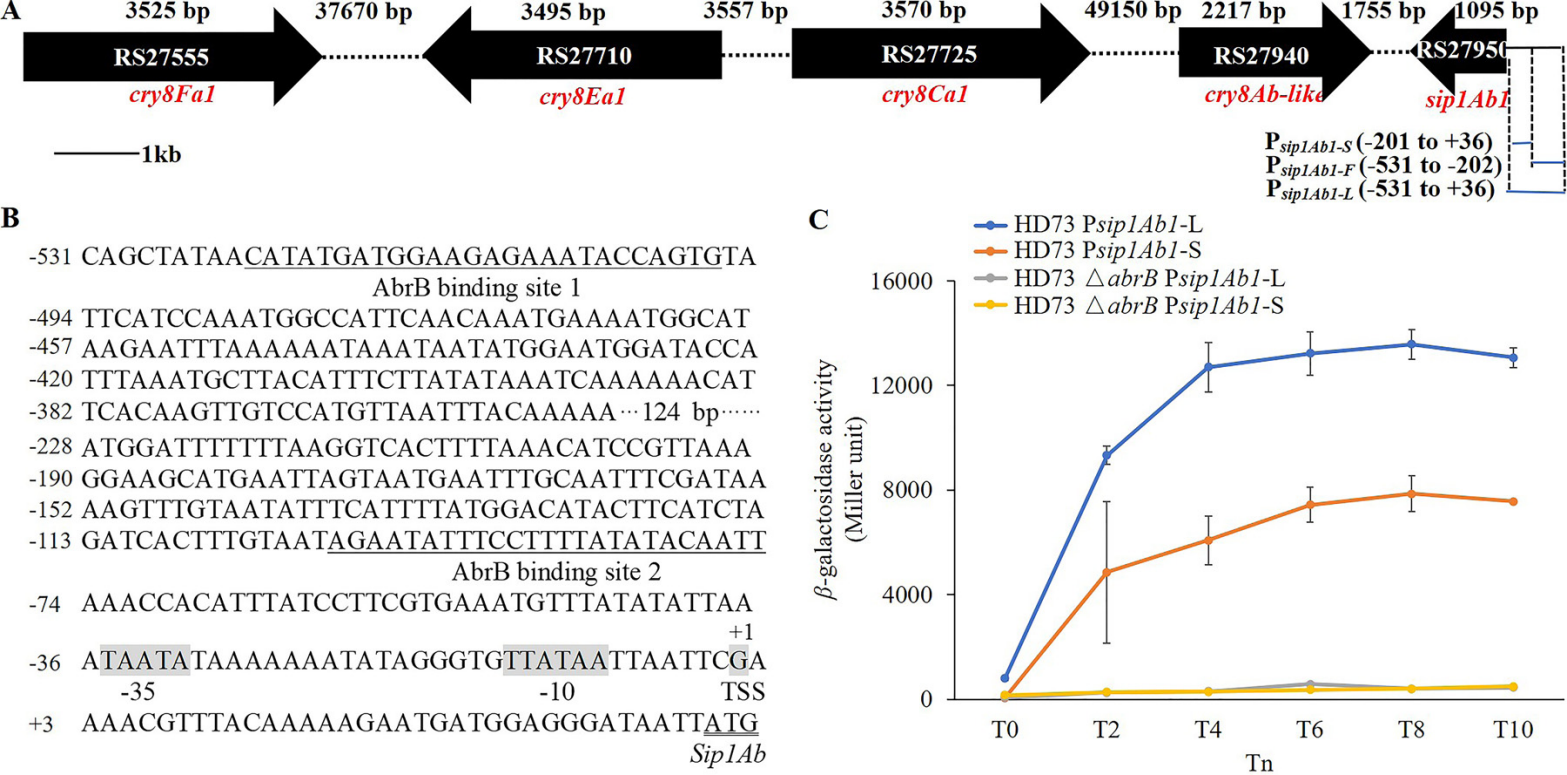
Transcriptional activity of *the sip1Ab1* promoter in B. thuringiensis HD73. (A) Map of the *cry* genes and *sip1Ab1* locus in Bacillus thuringiensis QZL38 plasmid 2 and construction of P*_sip1Ab1-L_* (−531 to +36), P*_sip1Ab1-F_* (−531 to −202) and P*_sip1Ab1-S_* (−201 to +36) promoters. Bar, 1 kb. Intergenic regions are not to scale. (B) Sequence analysis of 567 bp upstream of Bacillus thuringiensis QZL38 *sip1Ab1* ATG start codon. The transcription start site (TSS) and the putative −35 and −10 motifs are indicated with shades of gray. The two AbrB binding sites are indicated with underlines. (C) The activities of two *sip1Ab1* promoters (P*_sip1Ab1-L_* and P*_sip1Ab1-S_*) were assessed by *lacZ* fusions in HD73 and HD73 Δ*abrB* strains. Assays of β-galactosidase activity were performed to compare the activities of cells cultured in LB medium at 30°C with shaking at 220 rpm. *T*_0_ is the end of the exponential growth phase, and *T_n_* means *n* hours after *T*_0_. Each value represents the mean of at least three independent replicates. Error bars show the standard deviations.

Strain QZL38 contains 1 chromosome and 6 plasmids in, and the *sip1Ab1* gene (*RS27950*) is located on plasmid 2. The main *cry* genes include *cry8Ab-like*, *cry8Ca1*, *cry8Ea1*, and *cry8Fa1*, and their positions on plasmid 2 are shown in Fig. 2A. To determine the transcriptional start site of the *sip1Ab1* gene, a 5′ rapid amplification of cDNA ends (RACE)-PCR experiment was performed (see Materials and Methods). The transcriptional start site (TSS) was confirmed to be a G located 36 nucleotides upstream of the QZL38 *sip1Ab1* translational start codon (ATG) (Fig. 2B). The analysis of the QZL38 *sip1Ab1* promoter region contains −35 (TAATATAA) and −10 (TTATAATTA) regions from the transcriptional start site (Fig. 2B).

To clarify the transcriptional mechanism of *sip1Ab1*, the HD Δ*abrB* strain was constructed using the principle of homologous recombination. A P*_sip1Ab1-L_*-*lacZ* fusion was constructed and transformed into the B. thuringiensis HD73 and HD Δ*abrB* strains. The amplified fragment (P*_sip1Ab1-L_*) consisted of 567 bp upstream of the QZL38 *sip1Ab1* ATG start codon (Fig. 2A). The β-galactosidase activity assays (Fig. 2C) indicated that the transcriptional activities of P*_sip1Ab1-L_* in wild-type HD73 gradually increased between *T*_0_ to *T*_10_. The transcriptional activity of P*_sip1Ab1-L_* was significantly abolished in the HD Δ*abrB* strain compared to that in the wild-type HD73 strain. This indicates that deletion of the *abrB* gene inhibits the transcriptional activity of the *sip1Ab1* gene promoter. In other words, AbrB positively regulates the *sip1Ab1* gene.

### AbrB binds to the *sip1Ab1* promoter.

To test where AbrB directly regulates the *sip1Ab* gene, AbrB-His protein was purified via nickel column affinity chromatography. The desalting AbrB protein was dissolved in 20 mM Tris-HCl (pH 8.0).

**FIG 3:**
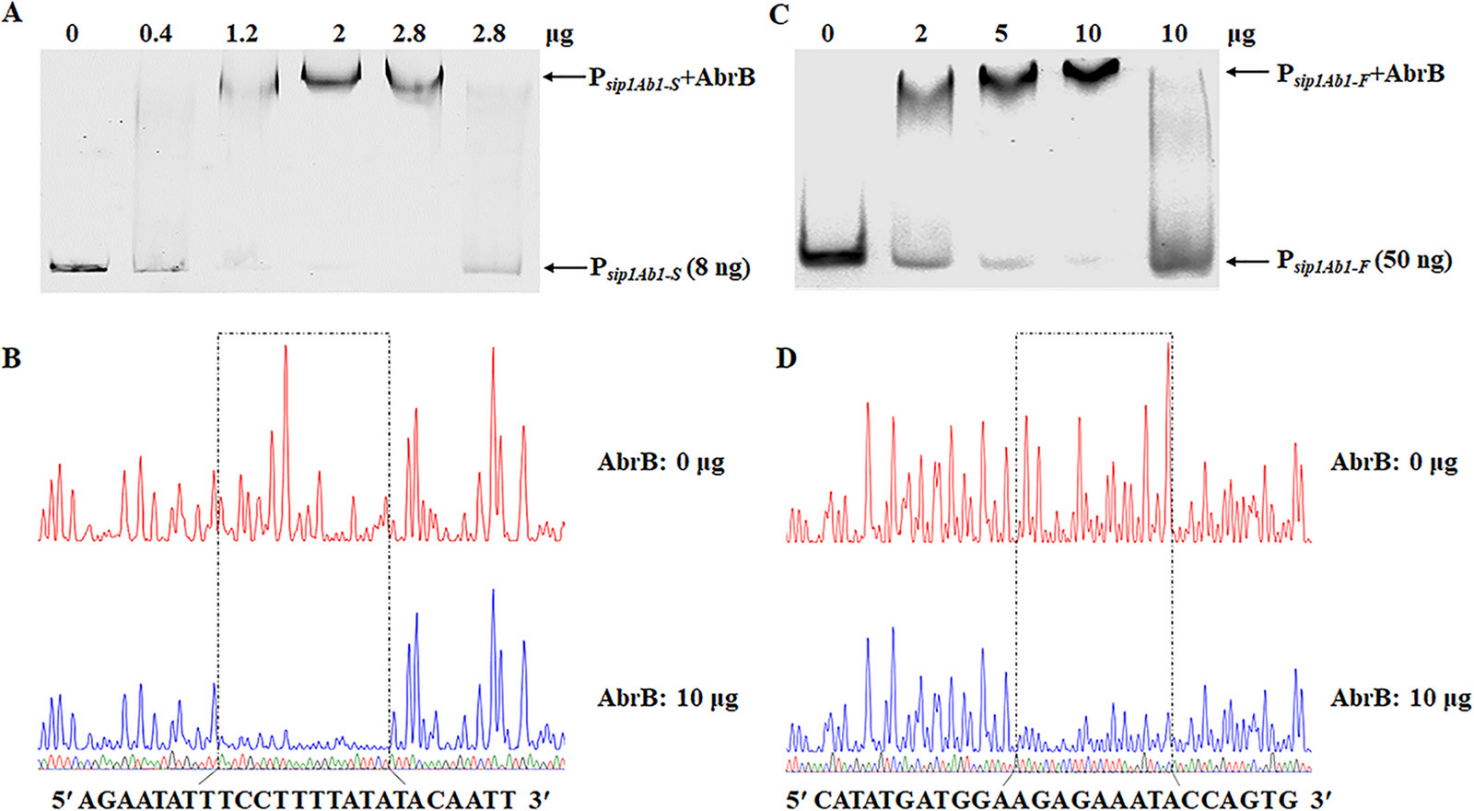
Identification of the AbrB protein binding sites in the *sip1Ab1* promoter. (A) Mobility shift assay of the *sip1Ab* promoter fragment *sip1Ab1-S* (−201 to +36) after interaction with AbrB protein. Lane 1, FAM-labeled P*_sip1Ab1-S_* probe; lanes 2 to 5, incubation of the probe with increasing concentrations of purified AbrB protein (indicated at the top of the figure); Lane 6, AbrB protein with P*_sip1Ab1-S_* probe and 200-fold unlabeled P*_sip1Ab1-S_*. Each lane contained 8 ng of probe. (B) Protection of a 25-bp sequence in the *sip1Ab1-S* promoter by AbrB, as revealed by a DNase I footprinting protection assay. The fluorograms correspond to the DNA in the protection reactions (with 0 and 10 μg AbrB). (C) Mobility shift assay of the *sip1Ab* promoter fragment *sip1Ab1-F* (−531 to −202) after interaction with AbrB protein. Lane 1, FAM-labeled P*_sip1Ab1-F_* probe; lanes 2 to 4, incubation of the probe with increasing concentrations of purified AbrB protein indicated at the top of the figure; lane 5, AbrB protein with P*_sip1Ab1-F_* probe and 200-fold unlabeled P*_sip1Ab1-F_*. Each lane contained 50 ng of probe. (D) Protection of a 26-bp sequence in the *sip1Ab1-F* promoter by AbrB, as revealed by a DNase I footprinting protection assay. The fluorograms correspond to the DNA in the protection reactions (with 0 and 10 μg AbrB).

A 567-bp promoter is too long for electrophoretic mobility shift assays (EMSA), so we first selected a region of the promoter (−201 to +36) (shown in Fig. 2A), P*_sip1Ab1-S_*, for EMSA. 6-carboxyfluorescein (FAM)-labeled fragments containing the promoter regions were incubated with increasing concentrations of AbrB. Protein-probe binding caused slow migration. Competitive gel shift assays were performed with labeled DNA probes and approximately 200-fold unlabeled DNA targets, respectively. As shown in Fig. 3A, 200-fold unlabeled DNA could dissociate most of the AbrB from the labeled promoter probe. The data shown in the figure confirm that AbrB directly binds to P*_sip1Ab1-S_* (Fig. 3A). To determine the AbrB binding site in the *sip1Ab1* promoter, a DNase I footprinting assay was performed using the same promoter fragment used in the EMSA. A fragment (5′-AGAATATTTCCTTTTATATACAATT-3′) of P*_sip1Ab1-S_* was protected via AbrB binding (Fig. 3B), corresponding to the underlined sequence in the *sip1Ab1* promoter region shown in Fig. 2B.

We compared the transcriptional activities of P*_sip1Ab1-S_* and P*_sip1Ab1-L_*. The results showed that the activity of P*_sip1Ab1-S_* was lower than that of P*_sip1Ab1-L_* in wild-type HD73, and it was also significantly abolished in the HD Δ*abrB* strain compared to that in the wild-type HD73 strain (Fig. 2C). We hypothesized that the promoter part P*_sip1Ab1-F_* (−531 to −202) may contain an AbrB binding site. The ability of AbrB to bind to P*_sip1Ab1-F_* was examined via EMSA. The data shown in Fig. 3 confirmed that AbrB directly binds to P*_sip1Ab1-F_* (Fig. 3C), and the DNase I footprinting assay showed that a fragment (5′-CATATGATGGAAGAGAAATACCAGTG-3′) of P*_sip1Ab1-F_* was protected via AbrB binding (Fig. 3D), corresponding to the underlined sequence in the *sip1Ab1* promoter region shown in Fig. 2B. This indicates that AbrB can bind with two regions of the *sip1Ab1* promoter and directly regulate the *sip1Ab1* gene.

**FIG 4:**
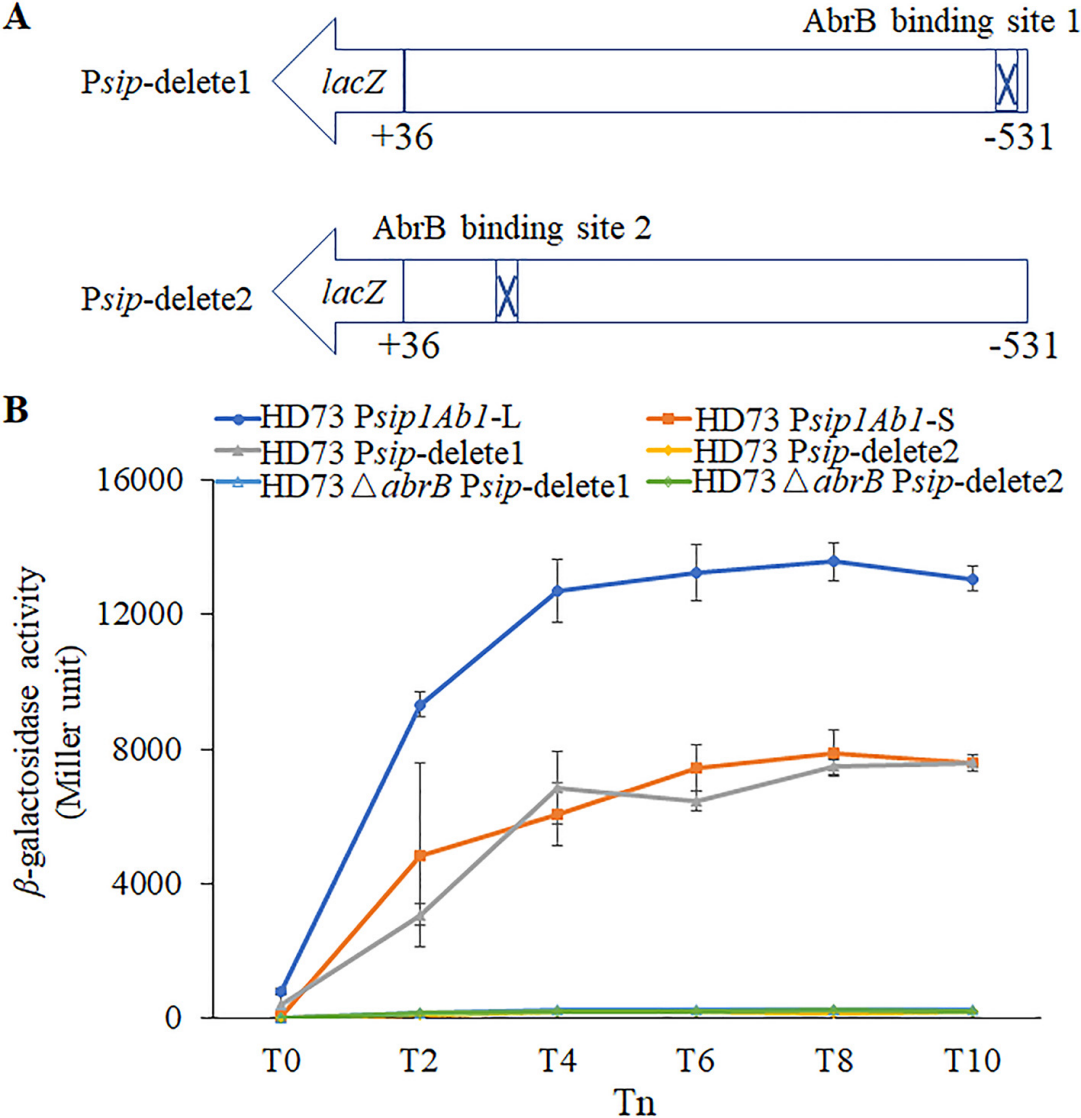
*sip1Ab1* promoter transcriptional activities. (A) Construction of P*sip*-delete1-*lacZ* and P*sip*-delete2-*lacZ*. (B) The activities of *sip1Ab1* promoters without binding site 1 or 2 (P*sip*-delete1 and P*sip*-delete2) were assessed by *lacZ* fusions in HD73 and HD73 Δ*abrB* strains. Assays of β-galactosidase activity were performed to compare the activities of cells cultured in LB medium at 30°C with shaking at 220 rpm. Each value represents the mean of at least three independent replicates. Error bars show the standard deviations.

To determine the roles of two AbrB binding sites, promoter fragments from ′531 to +36 from which binding site 1 or binding site 2 was deleted were fused to *lacZ* (Fig. 4A). The β-galactosidase activity assays (Fig. 4B) indicated that the transcriptional activity of P*_sip-_*delete1 in wild-type HD73 is no different from that of P*_sip1Ab1_*-S. The transcriptional activity of P*_sip-_*delete2 was very low. The transcriptional activity of P*_sip-_*delete1 was significantly abolished in the HD Δ*abrB* strain compared to that in the wild-type HD73 strain.

## DISCUSSION

Sip is a secreted protein, and Sip1Ab1 was secreted during the transition period (Fig. 1). Sporulation medium lacking nutrients has also been used to produce the Sip1Ab1 protein, but production was extremely low (data not shown). The Sip1Ab1 protein was secreted in sufficient nutrient medium (Fig. 1). Thus, nutrient-poor medium is not suitable for Sip1Ab1 protein expression, and medium that can extend the transition phase is suitable for Sip1Ab1 expression.

In this study, AbrB positively regulated the toxin gene in B. thuringiensis (Fig. 2C). AbrB mostly negatively regulates gene transcription, and it is considered to be a repressor of gene expression, although several genes were positively regulated by AbrB in B. subtilis. All previous studies showed that AbrB negatively regulates gene expression in the B. cereus group. In B. cereus, overexpression of AbrB resulted in a nontoxin phenotype, and the Spo0A-AbrB circuit negatively regulated *ces* and repressed cereulide production (27). In B. anthracis, AbrB negatively regulates toxin genes (24). In B. thuringiensis, AbrB repressed biofilm formation and motility, which showed similarities to those in B. subtilis (28). AbrB also negatively regulated the immune inhibitor metalloprotease *inhA1* (29). Currently, no toxin gene has been reported to be positively regulated by AbrB.

AbrB binds two regions of the *sip1Ab1* promoter, and the binding sequences were confirmed here (Fig. 3). P*_sip1Ab1-F_* and P*_sip1Ab1-S_* each have a binding sites, and the difference in activity indicates that the two sites have distinct roles. A previous study reported that AbrB bound to the *atxA* promoter in B. anthracis, and the binding sequence was identified (25). Since both B. thuringiensis and B. anthracis belong to the B. cereus group, the homology of AbrB between them is 99% (see Fig. S1 in the supplemental material). Thus, we performed an alignment of the AbrB binding sequences of B. thuringiensis and B. anthracis. A conserved DNA sequence that was analyzed by MEME, TTGKWTAWAAARGGAA, was identified (see Fig. S2 in the supplemental material). The conserved sequence is not consistent with the AbrB consensus binding sequence, which in B. subtilis consists of bipartite TGGNA motifs separated by 4 to 5 bp (30), but they were all extremely AT rich. As Koehler mentioned, the B. anthracis and B. subtilis AbrB binding sites are also somewhat different (31). Via AbrB protein sequence alignment analysis, it was found that B. anthracis and B. thuringiensis AbrB proteins are 85% identical to B. subtilis AbrB (Fig. S1), and the last 32 residues are significantly different (24). AbrB probably recognizes a three-dimensional DNA structure rather than a typical DNA sequence (13, 29, 32, 33). Here, we suggest that the AbrB consensus binding sequence in the B. cereus group is different from that in B. subtilis. Our results provide further understanding of the AbrB binding sequence and its regulatory mechanism.

## MATERIALS AND METHODS

### Strains, plasmids, and growth conditions.

The strains and plasmids used in this study are summarized in Table 1. Escherichia coli strains DH5α and BL21 were used as hosts for molecular cloning and protein expression, respectively. E. coli SCS110 (also called ET12567) was used for transformation into B. thuringiensis cells, as described previously (34). B. thuringiensis HD73 was used as the recipient strain to monitor gene transcriptional activity and manipulate the gene cloning of B. thuringiensis (35, 36). HD73 and its derivatives were routinely grown at 30°C in LB broth or on LB agar plates supplemented with either erythromycin (5 μg/ml) or kanamycin (100 μg/ml) when required.

**TABLE 1:** Strains and plasmids used in this study

Strain or plasmid	Characterization^*a*^	Source and/or reference
Strains		
Bacillus thuringiensis		
HD73	Bacillus thuringiensis subsp. *kurstaki*, carrying *cry1Ac* gene	Lab stock
HD (Δ*abrB*)	B. thuringiensis HD73 *abrB* gene insertion mutant; Kan^r^	This study
HD (*sip1Ab1*)	HD73 strain containing plasmid pHT*sip1Ab1*	This study
HD (P*_sip1Ab1-L_*)	HD73 strain containing plasmid pHTP*_sip1Ab1-L_*	This study
HD (P*_sip1Ab1-F_*)	HD73 strain containing plasmid pHTP*_sip1Ab1-F_*	This study
HD (P*_sip1Ab1-S_*)	HD73 strain containing plasmid pHTP*_sip1Ab1-S_*	This study
HD (P*_sip_*-delete1)	HD73 strain containing plasmid pHT P*_sip_*-delete1	This study
HD (P*_sip_*-delete2)	HD73 strain containing plasmid pHT P*_sip_*-delete2	This study
HD Δ*abrB* (P*_sip1Ab1-L_*)	HD (Δ*abrB*) strain containing plasmid pHTP*_sip1Ab1-L_*	This study
HD Δ*abrB* (P*_sip1Ab1-S_*)	HD (Δ*abrB*) strain containing plasmid pHTP*_sip1Ab1-S_*	This study
HD Δ*abrB* (P*_sip_*-delete1)	HD (Δ*abrB*) strain containing plasmid pHTP*_sip_*-delete1	This study
HD Δ*abrB* (P*_sip_*-delete2)	HD (Δ*abrB*) strain containing plasmid pHTP*_sip_*-delete2	This study
QZL38	B. thuringiensis carrying *cry8* genes and *sip1Ab1* gene	Lab stock
E. coli ET	F′ *dam-13*::Tn*9 dc*m-6 *hsdM hsdR recF143 zjj-202*::Tn*10 galK2 galT22 ara14 pacY1 xyl-5 leuB6 thi-1*; for generation of unmethylated DNA	Lab stock
Escherichia coli		
BL21	Escherichia coli	Lab stock
BL21 (pET*abrB*)	BL21 strain containing plasmid pET*abrB*	This study
BL21 (pET)	BL21 strain carrying pET21b	Lab stock
Plasmids		
pMAD	Amp^r^, Erm^r^ shuttle vector; thermosensitive origin of replication	Lab stock (40)
pMADΔ*abrB*	pMAD with *abrB* insertion fragment	This study
pHT304	Amp^r^, Erm^r^; E. coli-B. thuringiensis shuttle	Lab stock
pHT315	Amp^r^ Erm^r^; E. coli-B. thuringiensis shuttle	Lab stock
pET21b	Expressional vector; Amp^r^; 5.4 kb	Lab stock
pET*abrB*	pET21b containing *abrB* gene; Amp^r^	This study
pHTP*_sip1Ab1-L_*	pHT304-18Z carrying the promoter of *sip1Ab1*	This study
pHTP*_sip1Ab1-F_*	pHT304-18Z carrying half of the promoter of *sip1Ab1*	This study
pHTP*_sip1Ab1-S_*	pHT304-18Z carrying half of the promoter of *sip1Ab1*	This study
pHT*sip1Ab1*	pHT304 carrying *sip1Ab1* and P*_sip1Ab1-L_*	This study
pHT P*_sip_*-delete1	pHT304-18Z carrying the promoter of *sip1Ab1* with binding site 1 deleted	This study
pHT P*_sip_*-delete2	pHT304-18Z carrying the promoter of *sip1Ab1* with binding site 2 deleted	This study
pMAD19-TP*_sip1Ab1-F_*	pMAD19-T carrying half of the promoter of *sip1Ab1*	This study
pMAD19-TP*_sip1Ab1-S_*	pMAD19-T carrying half of the promoter of *sip1Ab1*	This study

a Kan^r^, kanamycin resistance; Amp^r^, ampicillin resistance; Erm^r^, erythromycin resistance.

### Secretion of Sip1Ab1 in QZL38 and HD73 strains.

A single B. thuringiensis colony was inoculated in 5 ml LB medium and grown at 30°C with shaking overnight, and then 1 ml of the bacterial solution was added to 100 ml of LB medium and incubated to *T*_0_ (*T*_0_ is the end of the exponential growth phase, and *T_n_* is *n* hours after the end of the exponential growth phase), *T*_6_, *T*_12_, and *T*_18_. A 50-ml aliquot of bacterial solution was collected and centrifuged (4°C at 9,000 rpm for 10 min), and then the supernatants were collected into the dialysis bag in each period. An appropriate amount of polyethylene glycol (PEG) 8000 was also needed outside the dialysis bag. The concentration time was generally 6 to 10 h, and the temperature was 4°C. The proteins were dissolved in 2 ml of 20 mM Tris-HCl, and the same volume was analyzed via SDS-PAGE. The protein bands were cut from the gel and sent to a company (Beijing Protein Innovation) for mass spectrometry identification. After the protein gel was decolorized, it was hydrolyzed under the action of trypsin and detected by mass spectrometer (MicrOTOF-Q11; Bruker Daltonics).

### Construction of the pHT*sip1Ab1* and pHTP*_sip_*-*lacZ* expression vectors.

The promoter sequences of the *sip1Ab1* gene were amplified from QZL38 genomic DNA (GenBank accession no. CP032609; region, 192552 to 193646) using different primers (Table 2). A 567-bp fragment located from −531 to +36 was PCR amplified from strain QZL38 with primers P*sip1Ab1-L-F* and P*sip1Ab1-L-R*, a 237-bp fragment located from −201 to +36 was PCR amplified from strain QZL38 with primers P*sip1Ab1-S-F* and P*sip1Ab1-L-R*, a 329-bp fragment located from −531 to −202 was PCR amplified from strain QZL38 with primers P*sip1Ab1-L-F* and P*sip1Ab1-F-R*, and a 553-bp/554-bp fragment which contained −531 to +36 except for binding site 1/binding site 2 was synthesized (Sangon Biotech, Shanghai) and ligated to the linearized pHT304-18Z plasmid, which contains a promoterless *lacZ* gene. A 1,598-bp fragment, containing the 567 bp upstream of the QZL38 *sip1Ab1* ATG start codon and the *sip1Ab1* open reading frame, was amplified and ligated to the linearized pHT304 plasmid. The recombinant plasmids were introduced into HD73. The resulting strains were placed on agar plates supplemented with erythromycin and verified by PCR.

**TABLE 2:** Sequences of oligonucleotide primers used in this study

Primer name	Oligonucleotide sequence (5′–3′) (restriction enzyme)^*a*^
P*_sip1Ab1-L_*-F	AA**CTGCAG**CAGCTATAACATATGATGGA (PstI)
P*_sip1Ab1-L_*-R	CG**GGATCC**AATTATCCCTCCATCATTC (BamHI)
P*_sip1Ab1-S_*-F	AA**CTGCAG**CATCCGTTAAAGGAAGCATGAATTAGTAATG (PstI)
P*_sip1Ab1-F_*-R	CG**GGATCC**TTTAAAAGTGACCTTAAAAAAATCC (BamHI)
*sip1Ab1*-F	AA**CTGCAG**CAGCTATAACATATGATGGA (PstI)
*sip1Ab1*-R	CG**GGATCC**TTAATTTCCACTTAAAATCTTTGTTTGAACAGG (BamHI)
304-F	CTATGACCATGATTACGCCAAGCTTGC
304-S	GGATGTGCTGCAAGGCGATTAAGTTGG
304-18Z F	CGTAATCTTACGTCAGTAACTTCCACAGTA
304-18Z R	CGCCAGGGTTTTCCCAGTCACGAC
*abrB*-a	TATCGATGCATGCCATGGTACCCGGGCATATGCCTGTGGCGTAATAT
*abrB*-b	CCTCAAATGGTTCGCTGTTTTAGATTCGTC ATTTTTCG
Km-F	CGAAAAATGACGAATCTAAAACAGCGAACCATTTGAGG
Km-R	GCTTATTTTG CTGTTTCGAT ATAAAATTCC TCGTAGGCGC
*abrB*-c	GCGCCTACGA GGAATTTTAT ATCGAAACAG CAAAATAAGC
*abrB*-d	TCGACGCGTC TGCAGAAGCT TCTAGAATTC TACGAGTTAT CATGAGCAC
AbrB-F	C**GGATCC**GATTATGAAATCTACTGGTATTG (BamHI)
AbrB-R	GC**GTCGAC**TTTTGCTGTTTCGATATAATCT(SalI)
JDWΔAbrB-F	GCCATGAGCAAAGACTTTCTTCGGA
JDWΔAbrB-R	GGTAGAGTGCTCTGATGGAAGTTAT
*sip1Ab1*5′race-R	CTTTAAATATCCAGAATCTAGCTAAAAACCATCC
P*_sip1-S_*-F	CATCCGTTAAAGGAAGCATGAATTA
P*_sip1-S_*-R	AATTATCCCTCCATCATTCTTTTTG
P*_sip1-F_*-F	CAGCTATAACATATGATGGAAGAGAAA
P*_sip1-F_*-R	TTTAAAAGTGACCTTAAAAAAATCC
M13F-FAM	GTAAAACGACGGCCAGT (5′ FAM labeled)
M13R-FAM	CAGGAAACAGCTATGAC (5 FAM labeled)

a FAM, 6-carboxyfluorescein. The restriction sites are underlined and bolded.

### β-Galactosidase activity analysis.

The B. thuringiensis *s*trains were cultivated in LB medium with shaking (220 rpm at 30°C). Aliquots (2 ml) of cultures were collected every 2-h interval from *T*_0_ to *T*_10_ (*T*_0_ indicates the end of the exponential growth phase, and *T_n_* indicates *n* hours after *T*_0_). The cells were centrifuged (13,000 × *g* for 1 min), and the pellets were stored at −20°C until use. *β*-Galactosidase activities were measured as previously described (37) and are expressed as Miller units. The reported values represent the averages from at least three independent assays.

### 5′ RACE analysis.

The total RNA was extracted from the QZL38 cells grown in LB until the *T*_3_ stage, and reverse transcription-PCR was conducted as previously described (38). We used the SMARTer RACE (switching mechanism at the 5′ end of the RNA transcript-rapid amplification of cDNA ends) cDNA amplification kit (Clontech, Mountain View, CA) to determine the transcription start site, following the manufacturer’s instructions. *sip1Ab1*5′race-R, located 200 bp downstream of the QZL38 *sip1Ab1* start codon (ATG), was designed as the specific reverse primer. NestRace was the forward primer provided in the kit (Clontech, Mountain View, CA). *sip1Ab1*5′race-R and NestRace were used as specific primers for amplifying the 5′ end of P*_sip1Ab1_* cDNA. The sequences of the primers used in this study are shown in Table 2.

### Screening of HD Δ*abrB* mutants.

The HD73 *abrB* mutant was constructed using the principle of homologous recombination. The method is briefly described as follows. A 709-bp upstream region containing a 15-bp overlap with the 5′ end of *abrB* (*abrB* fragment A) and a 729-bp downstream region containing a 21-bp overlap with the 3′ end of *abrB* (*abrB* fragment B) were amplified from B. thuringiensis HD73 genomic DNA with the *abrB*-a/*abrB-*b and *abrB*-c/*abrB*-d primer sets, respectively. The kanamycin resistance gene (*kan*, 1,473 bp) was amplified with the Km-F/Km-R primer set using the Δ*sigH* mutant as a template. Subsequently, a long flanking PCR was performed with *abrB* fragment A, the *kan* fragment, and *abrB* fragment B as the templates, in that order, and the *abrB*-a/*abrB*-d primer set to generate a long fragment (2,828 bp). The resulting DNA fragment was doubly digested with BamHI and SalI and cloned into the erythromycin (ERY)-resistant, temperature-sensitive suicide plasmid pMAD. The recombinant plasmid was named pMADΔ*abrB*. The recombinant plasmid was transferred to *E. coli* ET for demethylation and then electroporated into strain HD73. The strain was subjected to high-temperature mutation at 37°C, and a strain named HD (pMADΔ*abrB*), with kanamycin resistance and no erythromycin resistance, was selected. Using the mutant cassette outer primers JDwabrB-1 and JDwabrB-2, the wild-type strain HD73 and the kanamycin-resistant and ERY-resistant strain were used as the templates to identify the mutant strain, and the obtained mutant strain was named HD Δ*abrB*.

### Purification of the AbrB protein.

The BL21 (pET*abrB*) strain was cultured in LB medium containing 100 μg/ml ampicillin at 37°C and 220 r/min to an optical density at 600 nm (OD_600_) of 0.7 to 1.0 and then added to a final concentration of 0.5 mmol/liter isopropyl-β-d-thiogalactopyranoside (IPTG) induced at 18°C and 150 rpm for 12 h. The cells were collected by centrifugation at 9,000 rpm for 10 min at 4°C. The cells were suspended in 50 mM Tris-HCl buffer (pH 8.0). The suspension was ultrasonically disrupted on ice for 6 min (CP750, ultrasonic power 70%, ultrasound 3 s, and pause 5 s; Cole-Parmer). The supernatant and precipitate were separated by a low-temperature centrifuge (12,000 rpm for 10 min). The supernatant containing soluble AbrB protein was placed in a well-balanced nickel affinity chromatography column, and the His-tagged AbrB protein was fully combined with the column. Then, the protein was washed with 10 column volumes of equilibration buffer (20 mM/liter Tris-HCl [pH 8.0], 0.5 M/liter NaCl, and 20 mM/liter imidazole), and finally the proteins eluted by the 10 ml of elution buffer (20 mM/liter Tris-HCl [pH 8.0], 0.5 M/liter NaCl, and 250 mM/liter imidazole) were collected. SDS-PAGE was used to detect the eluted protein samples. The purified protein samples were desalted using the Äkta protein purifier, and the desalted protein was dissolved in 20 mM Tris-HCl (pH 8.0).

### Electrophoretic mobility shift assays.

The chosen gene of promoter sequences was amplified from QZL38 genomic DNA using different primers labeled with 6-carboxyfluorescein (FAM) (Table 2). The gel retardation assay determines the binding of the DNA fragment to the protein; 20 μl of the reaction system contains labeled DNA, different concentrations of AbrB protein and binding buffer [10 mM/liter Tris-HCl, 0.5 mM/liter dithiothreitol (DTT), 50 mM/liter NaCl, 500 ng poly(dI:dC) (pH 7.5), and 4% (vol/vol) glycerol]. The reaction was performed at 25°C for 30 min. The reaction product was detected via electrophoresis in 8% (wt/vol) nondenaturing polyacrylamide gel in TBE buffer (90 mM/liter Tris-base, 90 mM/liter boric acid, and 2 mmol/liter EDTA [pH 8.0]) (Mini-Protean system, 160 V, 4°C, 1 h; Bio-Rad). The nondenatured gel was scanned with a fluorescent gel imaging system (FLA Imager FLA-5100; laser, 473 nm; voltage, 900 V; filter, 526-000/01; Fujifilm).

### DNase I footprinting assay.

The promoter region was PCR amplified with 2× high-fidelity DNA polymerase premix (Tolo Biotech, Shanghai, China) from the plasmids pMAD19-TP*_sip1Ab1-F_* and pMAD19-TP*_sip1Ab1-S_* using M13F (FAM) and M13R primers to prepare the fluorescent FAM-labeled probes. The FAM-labeled probes were purified using the Wizard SV gel and PCR clean-up system (Promega, USA) and were quantified with a NanoDrop 2000C instrument (Thermo, USA). The DNase I footprinting assays were performed following Wang et al. (39).

## References

[1] Vilas-Bôas GT, Peruca AP, Arantes OM. 2007. Biology and taxonomy of *Bacillus cereus*, *Bacillus anthracis*, and *Bacillus thuringiensis*. Can J Microbiol 53:673–687. doi:10.1139/W07-029. 17668027

[2] Ehling-Schulz M, Lereclus D, Koehler TM. 2019. The *Bacillus cereus* group: *Bacillus* species with pathogenic potential. Microbiol Spectr 7. doi:10.1128/microbiolspec.GPP3-0032-2018. PMC6530592 31111815

[3] Beegle CC, Yamamoto T. 1992. Invitation paper (C.P. Alexander Fund): history of *Bacillus thuringiensis* Berliner research and development. Can Entomol 124:587–616. doi:10.4039/Ent124587-4.

[4] Jouzani GS, Valijanian E, Sharafi R. 2017. *Bacillus thuringiensis*: a successful insecticide with new environmental features and tidings. Appl Microbiol Biotechnol 101:2691–2711. doi:10.1007/s00253-017-8175-y. 28235989

[5] Chattopadhyay P, Banerjee G. 2018. Recent advancement on chemical arsenal of *Bt* toxin and its application in pest management system in agricultural field. 3 Biotech 8:201. doi:10.1007/s13205-018-1223-1. PMC5874219 29607282

[6] Baranek J, Kaznowski A, Konecka E, Naimov S. 2015. Activity of vegetative insecticidal proteins Vip3Aa58 and Vip3Aa59 of *Bacillus thuringiensis* against lepidopteran pests. J Invertebr Pathol 130:72–81. doi:10.1016/j.jip.2015.06.006. 26146224

[7] Palma L, Muñoz D, Berry C, Murillo J, Caballero P. 2014. Draft genome sequences of two *Bacillus thuringiensis* strains and characterization of a putative 41.9-kDa insecticidal toxin. Toxins (Basel) 6:1490–1504. doi:10.3390/toxins6051490. 24784323 PMC4052248

[8] Donovan WP, Engleman JT, Donovan JC, Baum JA, Bunkers GJ, Chi DJ, Clinton WP, English L, Heck GR, Ilagan OM, Krasomil-Osterfeld KC, Pitkin JW, Roberts JK, Walters MR. 2006. Discovery and characterization of Sip1A: a novel secreted protein from *Bacillus thuringiensis* with activity against coleopteran larvae. Appl Microbiol Biotechnol 72:713–719. doi:10.1007/s00253-006-0332-7. 16489451

[9] Sha J, Zhang J, Chi B, Liu R, Li H, Gao J. 2018. *Sip1Ab* gene from a native *Bacillus thuringiensis* strain QZL38 and its insecticidal activity against *Colaphellus bowringi* Baly. Biocontrol Sci Techn 28:459–467. doi:10.1080/09583157.2018.1460313.

[10] Nielsen H, Engelbrecht J, Brunak S, von Heijne G. 1997. Identification of prokaryotic and eukaryotic signal peptides and prediction of their cleavage sites. Protein Eng 10:1–6. doi:10.1093/protein/10.1.1. 9051728

[11] Palma L, Muñoz D, Berry C, Murillo J, Caballero P. 2014. *Bacillus thuringiensis* toxins: an overview of their biocidal activity. Toxins (Basel) 6:3296–3325. doi:10.3390/toxins6123296. 25514092 PMC4280536

[12] Strauch MA, Perego M, Burbulys D, Hoch JA. 1989. The transition state transcription regulator AbrB of *Bacillus subtilis* is autoregulated during vegetative growth. Mol Microbiol 3:1203–1209. doi:10.1111/j.1365-2958.1989.tb00270.x. 2507867

[13] Strauch MA, Spiegelman GB, Perego M, Johnson WC, Burbulys D, Hoch JA. 1989. The transition state transcription regulator *abrB* of *Bacillus subtilis* is a DNA binding protein. EMBO J 8:1615–1621. doi:10.1002/j.1460-2075.1989.tb03546.x. 2504584 PMC400994

[14] Chumsakul O, Takahashi H, Oshima T, Hishimoto T, Kanaya S, Ogasawara N, Ishikawa S. 2011. Genome-wide binding profiles of the *Bacillus subtilis* transition state regulator AbrB and its homolog Abh reveals their interactive role in transcriptional regulation. Nucleic Acids Res 39:414–428. doi:10.1093/nar/gkq780. 20817675 PMC3025583

[15] Bobay BG, Benson L, Naylor S, Feeney B, Clark AC, Goshe MB, Strauch MA, Thompson R, Cavanagh J. 2004. Evaluation of the DNA binding tendencies of the transition state regulator AbrB. Biochemistry 43:16106–16118. doi:10.1021/bi048399h. 15610005

[16] Kim HJ, Kim SI, Ratnayake-Lecamwasam M, Tachikawa K, Sonenshein AL, Strauch M. 2003. Complex regulation of the *Bacillus subtilis* aconitase gene. J Bacteriol 185:1672–1680. doi:10.1128/JB.185.5.1672-1680.2003. 12591885 PMC148081

[17] Strauch MA, Hoch JA. 1993. Transition-state regulators: sentinels of *Bacillus subtilis* post-exponential gene expression. Mol Microbiol 7:337–342. doi:10.1111/j.1365-2958.1993.tb01125.x. 8459762

[18] Lozano Goné AM, Dinorín Téllez Girón J, Jiménez Montejo FE, Hidalgo-Lara ME, López Y López VE. 2014. Behavior of transition state regulator AbrB in batch cultures of *Bacillus thuringiensis*. Curr Microbiol 69:725–732. doi:10.1007/s00284-014-0650-4. 25002359

[19] Ma W, Peng D, Walker SL, Cao B, Gao CH, Huang Q, Cai P. 2017. *Bacillus subtilis* biofilm development in the presence of soil clay minerals and iron oxides. NPJ Biofilms Microbiomes 3:4. doi:10.1038/s41522-017-0013-6. 28649405 PMC5445608

[20] Stein T. 2020. Oxygen-limiting growth conditions and deletion of the transition state regulator protein AbrB in *Bacillus subtilis* 6633 result in an increase in subtilosin production and a decrease in subtilin production. Probiotics Antimicrob Proteins 12:725–731. doi:10.1007/s12602-019-09547-4. 30980290

[21] Barbieri G, Albertini AM, Ferrari E, Sonenshein AL, Belitsky BR. 2016. Interplay of CodY and ScoC in the regulation of major extracellular protease genes of *Bacillus subtilis*. J Bacteriol 198:907–920. doi:10.1128/JB.00894-15. 26728191 PMC4772597

[22] Zheng C, Yu Z, Du C, Gong Y, Yin W, Li X, Li Z, Römling U, Chou SH, He J. 2020. 2-Methylcitrate cycle: a well-regulated controller of *Bacillus* sporulation. Environ Microbiol 22:1125–1140. doi:10.1111/1462-2920.14901. 31858668

[23] Strauch MA. 1995. AbrB modulates expression and catabolite repression of a *Bacillus subtilis* ribose transport operon. J Bacteriol 177:6727–6731. doi:10.1128/jb.177.23.6727-6731.1995. 7592460 PMC177535

[24] Saile E, Koehler TM. 2002. Control of anthrax toxin gene expression by the transition state regulator *abrB*. J Bacteriol 184:370–380. doi:10.1128/JB.184.2.370-380.2002. 11751813 PMC139583

[25] Strauch MA, Ballar P, Rowshan AJ, Zoller KL. 2005. The DNA-binding specificity of the *Bacillus anthracis* AbrB protein. Microbiology (Reading) 151:1751–1759. doi:10.1099/mic.0.27803-0. 15941984

[26] Yang H, Wang P, Peng Q, Rong R, Liu C, Lereclus D, Zhang J, Song F, Huang D. 2012. Weak transcription of the *cry1Ac* gene in nonsporulating *Bacillus thuringiensis* cells. Appl Environ Microbiol 78:6466–6474. doi:10.1128/AEM.01229-12. 22773626 PMC3426696

[27] Lücking G, Dommel MK, Scherer S, Fouet A, Ehling-Schulz M. 2009. Cereulide synthesis in emetic *Bacillus cereus* is controlled by the transition state regulator AbrB, but not by the virulence regulator PlcR. Microbiology (Reading) 155:922–931. doi:10.1099/mic.0.024125-0. 19246763

[28] Fagerlund A, Dubois T, Økstad OA, Verplaetse E, Gilois N, Bennaceur I, Perchat S, Gominet M, Aymerich S, Kolstø AB, Lereclus D, Gohar M. 2014. SinR controls enterotoxin expression in *Bacillus thuringiensis* biofilms. PLoS One 9:e87532. doi:10.1371/journal.pone.0087532. 24498128 PMC3909190

[29] Grandvalet C, Gominet M, Lereclus D. 2001. Identification of genes involved in the activation of the *Bacillus thuringiensis inhA* metalloprotease gene at the onset of sporulation. Microbiology (Reading) 147:1805–1813. doi:10.1099/00221287-147-7-1805. 11429458

[30] Onuma C, Kensuke N, Tetsuya K, Tomoaki S, Hobman JL, Naotake O, Taku O, Shu I. 2013. High-resolution mapping of *in vivo* genomic transcription factor binding sites using *in situ* DNase I footprinting and ChIP-seq. DNA Res 20:325–338. doi:10.1093/dnares/dst013. 23580539 PMC3738160

[31] Koehler TM. 2002. *Bacillus anthracis* genetics and virulence gene regulation. Curr Top Microbiol Immunol 271:143–164. doi:10.1007/978-3-662-05767-4_7. 12224521

[32] Olson AL, Tucker AT, Bobay BG, Soderblom EJ, Moseley MA, Thompson RJ, Cavanagh J. 2014. Structure and DNA-binding traits of the transition state regulator AbrB. Structure 22:1650–1656. doi:10.1016/j.str.2014.08.018. 25308864 PMC4252516

[33] Xu K, Strauch MA. 1996. *In vitro* selection of optimal AbrB-binding sites: comparison to known in vivo sites indicates flexibility in AbrB binding and recognition of three-dimensional DNA structures. Mol Microbiol 19:145–158. doi:10.1046/j.1365-2958.1996.358882.x. 8821944

[34] Chen X, Gao T, Peng Q, Zhang J, Chai Y, Song F. 2018. Novel cell wall hydrolase CwlC from *Bacillus thuringiensis* is essential for mother cell lysis. Appl Environ Microbiol 84:e02640-17. doi:10.1128/AEM.02640-17. 29374039 PMC5861822

[35] Du C, Nickerson KW. 1996. *Bacillus thuringiensis* HD-73 spores have surface-localized Cry1Ac toxin: physiological and pathogenic consequences. Appl Environ Microbiol 62:3722–3726. doi:10.1128/aem.62.10.3722-3726.1996. 16535421 PMC1388959

[36] Liu G, Song L, Shu C, Wang P, Deng C, Peng Q, Lereclus D, Wang X, Huang D, Zhang J, Song F. 2013. Complete genome sequence of *Bacillus thuringiensis* subsp. *kurstaki* strain HD73. Genome Announc 1:e0008013. doi:10.1128/genomeA.00080-13. 23516207 PMC3622971

[37] Peng Q, Liu C, Wang B, Yang M, Wu J, Zhang J, Song F. 2016. Sox transcription in sarcosine utilization is controlled by sigma^54^ and SoxR in *Bacillus thuringiensis* HD73. Sci Rep 6:29141. doi:10.1038/srep29141. 27404799 PMC4941409

[38] Du L, Qiu L, Peng Q, Lereclus D, Zhang J, Song F, Huang D. 2012. Identification of the promoter in the intergenic region between *orf1* and *cry8Ea1* controlled by sigma H factor. Appl Environ Microbiol 78:4164–4168. doi:10.1128/AEM.00622-12. 22504821 PMC3370531

[39] Wang Y, Cen XF, Zhao GP, Wang J. 2012. Characterization of a new GlnR binding box in the promoter of *amtB* in *Streptomyces coelicolor* inferred a PhoP/GlnR competitive binding mechanism for transcriptional regulation of *amtB*. J Bacteriol 194:5237–5244. doi:10.1128/JB.00989-12. 22821977 PMC3457235

[40] Arnaud M, Chastanet A, Debarbouille M. 2004. New vector for efficient allelic replacement in naturally nontransformable, low-GC-content, Gram-positive bacteria. Appl Environ Microbiol 70:6887–6891. doi:10.1128/AEM.70.11.6887-6891.2004. 15528558 PMC525206

