# Supplementary material for: Transition Phase Regulator AbrB Positively Regulates the sip1Ab1 Gene Expression in Bacillus thuringiensis
Source: Microbiol Spectr. 2021 Jul 28;9(1):10.1128/spectrum.00075-21. doi: 10.1128/spectrum.00075-21 (PMC8552724; doi:10.1128/spectrum.00075-21)
Supplement: SUPPLEMENTAL FILE 1 — Supplemental material. Download SPECTRUM00075-21_Supp_1_seq2.docx, DOCX file, 0.2 MB [file spectrum00075-21_supp_1_seq2.docx]

**Transition phase regulator AbrB positively regulates the *sip1Ab1* gene in *Bacillus thuringiensis***

Xinxin Shen^a, b^, Qingyue Yu^b^, Huanhuan Liu^a, b^, Jiaojiao Wang^b^, Ruibin Zhang^b^, Qi Peng^b^, Fuping Song^a, b^#

^a^College of Life Science, Northeast Agricultural University, Harbin, China.

^b^State Key Laboratory for Biology of Plant Diseases and Insect Pests, Institute of Plant Protection, Chinese Academy of Agricultural Sciences, Beijing, China.

#Corresponding author Fuping Song: [fpsong@ippcaas.cn](mailto:fpsong@ippcaas.cn)


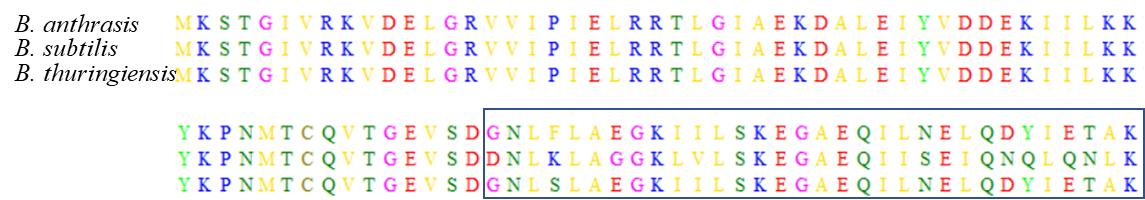


Fig. S1 Aligment of AbrB protein sequence of *B. anthrasis*, *B. subtilis* and *B. thuringiensis*. Homology of AbrB between *B. anthrasis* and *B. thuringiensis* is 99%. *B. anthrasis* and *B. thuringiensis* AbrB are 85% identical to *B. subtilis* AbrB, the first 62 Aa are exactly the same, the last 32 residues (square circle) are different significantly, only 56% identity to *B. subtilis.*


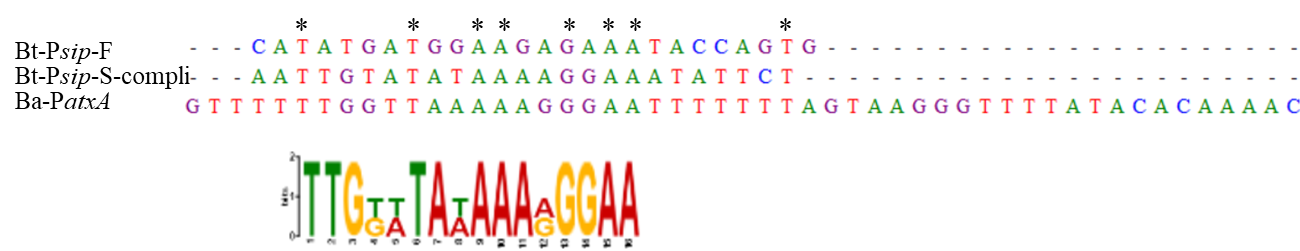


Fig. S2 Aligment of AbrB binding sequences and characterization of a conserved DNA sequence by MEME.
